# Multi-scale analysis of habitat fragmentation on small-mammal abundance and tick-borne pathogen infection prevalence in Essex County, MA

**DOI:** 10.1371/journal.pone.0269768

**Published:** 2022-06-13

**Authors:** Samuel D. Mason, Samuel C. R. Sherratt, Samantha M. Kruguer, Michael Muthersbaugh, Jonathan P. Harris, Wayne C. Gatlin, Justin D. Topp, Gregory S. Keller

**Affiliations:** 1 Landscape Ecology Lab, Department of Biology, Gordon College, Wenham, Massachusetts, United States of America; 2 Department of Integrative Biology, Oklahoma State University, Stillwater, Oklahoma, United States of America; 3 Department of Biology, Endicott College, Beverly, Massachusetts, United States of America; Tufts University Cummings School of Veterinary Medicine, UNITED STATES

## Abstract

Habitat fragmentation and heterogeneity transform otherwise contiguous tracks of forest into smaller patches in the northeastern U.S. and likely impact abundances, movement patterns, and disease transmission pathways for small-mammal communities at multiple scales. We sought to determine the structure of a small-mammal community in terms of mammal abundance and infection prevalence of *Borrelia burgdorferi* sensu stricto (s.s.), *Anaplasma phagocytophilum*, and *Babesia microti* within a fragmented landscape in Essex County, Massachusetts, USA. We studied communities at multiple spatial scales, including vegetation, edge type, and landscape (including 200-m, 500-m, and 1000-m radii) scales. A total of 16 study sites were chosen to represent four edge types: interior forest, pasture edge, natural edge, and residential edge. At each site, we trapped small mammals and conducted vegetation surveys and GIS analysis. Upon capture, a tissue sample was collected to analyze for presence of pathogens. Northern short-tailed shrew (*Blarina brevicauda*) abundance did not differ based on edge type, whereas abundance of the white-footed mouse (*Peromyscus leucopus*) was greatest at pasture edges, although the relationship was relatively weak. White-footed mouse abundance was negatively associated with amount of forested area within a 500-m radius, whereas northern short-tailed shrew abundance demonstrated a positive relationship with fragmentation indices at the 200-m radius. White-footed mice captured at interior-forest habitat were more likely be infected with *B*. *burgdorferi* (s.s.) than individuals from edge habitat. Greater prevalence of *B*. *burgdorferi* infection of white-footed mice in forest interiors compared to edge habitats counters previous studies. Reasons for this and implications are discussed.

## Introduction

Habitat fragmentation and loss are pervasive challenges to biological populations in New England, USA. Increases in agricultural, residential, and commercial development over the past four decades have led to consistent reductions in forested area in northern Massachusetts, with approximately 63% of the state remaining forested [1]. Forest continuity is negatively associated with human population density [2], with more densely populated townships of northeastern Massachusetts characterized by small patch size (< 10 ha) and low proportions of core habitat relative to higher proportions of edge habitat [3].

The effects of habitat fragmentation on small mammals have been widely studied, but no unidirectional trends in abundance have been well-established [4]. However, species-specific demographic trends tend to generate a consensus fragmentation effect. For example, Manson et al. (1999) [5] demonstrated higher capture probabilities at forest edges for white-footed mice (*Peromyscus leucopus*), whereas Glennon and Porter (2007) [6] found shrews of the *Sorex* genus prefer contiguous landscapes with minimal edge habitat. White-footed mice also may be more common in smaller than larger forest patches [7]. These differential associations are commonly ascribed to the life histories of each species, where generalists capable of acquiring resources from both the habitat patch and the matrix are able to thrive in more fragmented landscapes [4, 8].

Members of small-mammal communities serve as significant reservoirs in pathogen transmission. In New England, *Borrelia burgdorferi* sensu stricto (hereafter *B*. *burgdorferi*) (Lyme disease), *Babesia microti* (human babesiosis), and *Anaplasma phagocytophilum* (human granulocytic anaplasmosis) are the most prevalent tick-borne pathogens and are transmitted by their common vector, the black-legged tick (*Ixodes scapularis*) [9–12]. Although many small-mammal species carry these pathogens, the white-footed mouse and northern short-tailed shrew (*Blarina brevicauda*) are common members of the New England small-mammal community and serve as reservoirs for all three pathogens [9, 12–15]. Research has demonstrated that *B*. *burgdorferi* and *B*. *microti* are more prevalent within forest interiors (50 m from an edge) than forest edges in *Ixodes scapularis* [16] and at large natural sites compared to more fragmented sites in *Ixodes ricinus* [17]. Furthermore, habitat fragmentation is positively associated with both black-legged tick density and *B*. *burgdorferi* infection prevalence, indicating that the core habitat (interiors) of patchy landscapes may represent high-risk zones for Lyme disease transmission [18, 19]. Neither *B*. *burgdorferi* nor *A*. *phagocytophilum* prevalence appear to respond to changes in fragmentation along an urbanization gradient [20], and the effect of habitat fragmentation on movement patterns of *A*. *phagocytophilum* reservoirs remains unclear [9, 21].

In this study, we sought to determine the abundance of a small-mammal community and determine the potential of *Peromyscus* species and the northern short-tailed shrew as reservoirs of *B*. *burgdorferi*, *A*. *phagocytophilum*, and *B*. *microti* within a fragmented landscape in Essex County, Massachusetts, USA. Specifically, we considered how differences in fragmentation and habitat characteristics were related to abundance and infection prevalence of small mammals. We analyzed study sites in terms of vegetation composition, edge type, and landscape variation at 200 m, 500 m, and 1000 m. Based on the literature, we predicted that white-footed mice would be more abundant in natural, pasture, and residential edges and also negatively influenced by patch size, whereas northern short-tailed shrews would be more abundant in interior forest and negatively affected by habitat fragmentation. We also hypothesized that infection prevalence of small mammals would be positively related to fragmentation at multiple spatial scales and with greater understory vegetation.

## Materials and methods

### Study sites and site selection

This study is part of a larger research effort on differential habitat use by terrestrial vertebrates affected by edge type, landscape composition, and vegetation structure in northeastern Massachusetts [22]. Essex County is dominated by both historic and recent residential development, agricultural fields, and pastureland for horses, with relatively flat, low-elevation (30–75 m) topography. The study area is bounded by the UTM coordinates: 19N 345818E 4726418N; 355972E 4726418N; 355972E 4713148N; 345818E 4713148N. Within this area, we identified public lands that were accessible during the summer and randomly selected sites for each habitat category using ArcMap 9.3 [23]. Sites were ground-truthed to determine suitability and extent of human activity. Permission was granted by land owners; permits were not required for access.

Common overstory trees included white pine (*Pinus strobus*), eastern hemlock (*Tsuga canadensis*), oak (*Quercus* spp.), red maple (*Acer rubrum*), American beech (*Fagus grandifolia*), birch (*Betula* spp.), shagbark hickory (*Carya ovata*), horse chestnut (*Aesculus hippocastanum*), and cherry (*Prunus* spp.). Understory was dominated by seedlings and saplings of overstory trees, in addition to Japanese barberry (*Berberis thunbergii*), Japanese honeysuckle (*Lonicera japonica*), multiflora rose (*Rosa multiflora*), flowering dogwood (*Cornus florida*), poison ivy (*Toxicodendron radicans*), sassafras (*Sassafras albidum*), and viburnam (*Viburnum spp*.). We selected sites that appeared to be similar in vegetation structure and tree species composition, and site characteristics did not appear to change between years. Sites were placed in mature forest (greater than 40 years old) at areas free from active disturbance (e.g., cattle grazing, cutting) in forest patches at least 10 ha in size.

**Fig 1:**
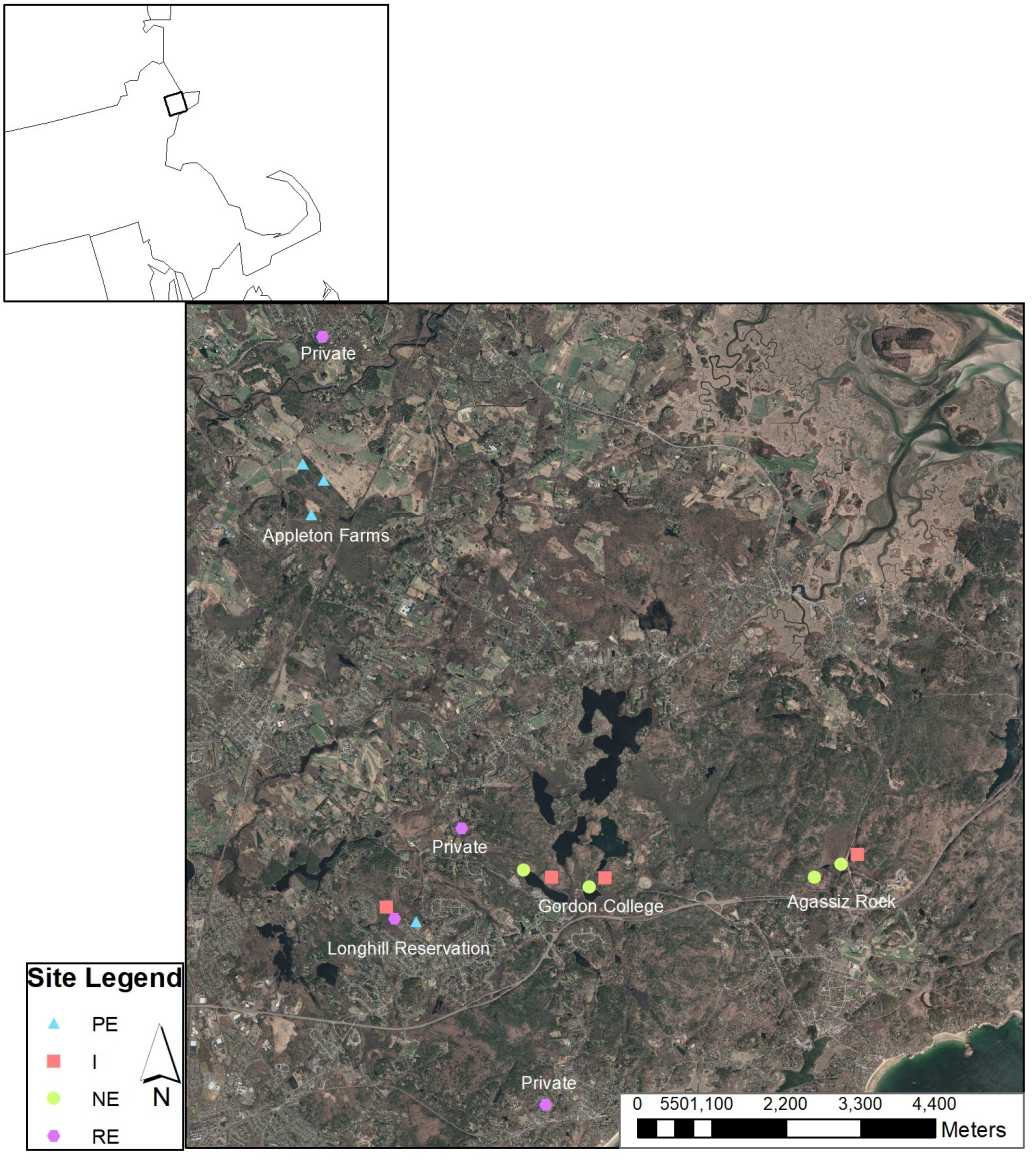
Site locations for small-mammal trapping in northeastern Massachusetts, Essex County, USA, for pasture-edge, natural-edge, residential-edge, and interior-forest sites.

We surveyed 16 study sites in southwestern Essex County MA, with four sites each in four edge types: interior-forest, pasture-edge, natural-edge, and residential-edge habitats (Fig 1). Edge sites were placed within 20 m of the interface between forested habitat and undeveloped open lands (e.g. pastures) for pasture-edge, bodies of water (e.g. wetlands and ponds) for natural-edge, and residential developments (e.g. neighborhoods) for residential-edge site, respectively. Interior-forest sites were located within a forest patch at least 150 m from an edge. We established sites at least 300 m apart from one another to maintain site independence.

### Small-mammal surveys

Research followed American Society of Mammalogists guidelines [24] and was approved by the Gordon College Internal Review Board. Each of the 16 study sites was surveyed for small mammals using 7.5 x 8.7 x 30 cm non-folding aluminum Sherman traps baited with rolled oats and peanut butter; cotton balls were added for bedding. Fifty traps were set at each site for 3 nights during 2012 and 2013 (September to early October 2012 and late July to August 2013), yielding a total of 4800 trap nights. Each site was trapped for only one 3-night session each year. Within a study site, 25 traps were set every 3 m along 2 parallel 75-m transects, which were established 3 m apart from each other. Trap lines were located parallel to the forest-edge interface (where applicable) in mixed-forest habitat. Traps were typically set in the late afternoon and checked daily for 3 consecutive mornings.

Upon capture, all small mammals were identified to the species level (except for members of the *Peromyscus* genus, which could not be distinguished accurately morphologically), weighed, tagged with aluminum tags, sampled by taking a small ear clipping or tail clipping (shrews), and released. Tissue samples were stored in 70% ethanol and collected for genetic species discrimination between *Peromyscus* spp. and for assessment of tick-borne pathogen infection among all small mammals.

### Vegetation surveys and landscape analysis

We conducted vegetation surveys at sites from June to July 2009 and during early September 2013. We adapted our vegetation survey protocol from James and Shugart (1970) [25], following the methods of Keller et al. (2009) [26], by including variables likely to be influential in small-mammal communities. Surveys were conducted within a 12-m radius from the center of the study site as a representation of the vegetation at the site. Within this circle, we established four 1x12-m transects in the cardinal directions. Along these transects, the number of understory trees (2.5–7.5 cm diameter at breast height [DBH], > 1.5 m height), short shrubs (< 2.5 cm DBH, 0.5–1.5 m height), and tall shrubs (< 2.5 cm DBH, > 1.5 m height) were counted. We also determined percentage of canopy cover, bare ground, dead ground, herbaceous ground, and leaf litter at each site every 2 m from the center of the site along each of the cardinal directions to yield a total of 20 measurements (X 5% = 100%). Within the 12-m radius circle, we measured DBH and recorded to genus all overstory trees (> 7.5 cm DBH and > 1.5 m height). From these values, basal area for an individual tree was calculated as π(DBH/2)^2^. These values were summed to determine total basal area (TBA) of deciduous and coniferous trees separately as a measure of biomass. Within the 12-m radius, we also counted total number of logs (> 7.5 cm DBH, > 1 m in length) and snags (standing dead overstory trees).

Using ArcMap 9.3 (ESRI 2009) [23], we produced a GIS of the 16 study sites from 15-cm resolution digital orthophoto quarterquads (DOQQs) satellite imagery of Essex County (downloaded from http://maps.massgis.state.ma.us/map_ol/oliver.php). We measured four landscape metrics at each of the 16 study sites, including patch size (total area of the patch within which the study site occurred, separated by major roadways or other inhospitable habitat from other patches), forested area, linear human edge (length of edge occurring at the interface between human-developed land and forest habitat), and linear wetland edge (length of edge occurring at the interface between a body of water and forest habitat). Other than forest patch size, these landscape variables were measured within three radii, including 200, 500 and 1000 m, representing distances above the home-range size and incorporating maximum long-distance dispersal movements for species we captured [27, 28]. These radii also overlapped with previous analyses of habitat use patterns in small mammals [29]. Within each radius we summed the areas of the delineated forest polygons and the lengths of the human and natural edge polylines to estimate forested area and amount of linear human and natural edge. To better represent fragmentation within the landscape, we calculated a fragmentation index for each radius by dividing the length of induced edge (edge produced through fragmentation) by the total forested area of that site at the same radius.

### DNA extraction and infection analyses

DNA was extracted from small-mammal tissue samples using the Qiagen DNeasy^®^ Blood and Tissue kit (Cat. No. 69504, Qiagen, Germantown, MD) following the manufacturer’s recommended protocol and eluting in 50 ul H_2_O. Gel electrophoresis and concentration by NanoDrop (ThermoFisher) were used to assess DNA purity and quantity. Samples containing sufficient amounts of DNA for analysis were subjected to multiplex qPCR for detection of *B*. *burgdorferi* (gB31), *A*. *phagocytophilum* (msp4), and *B*. *microti* (18S) in a 96-well format using the methods developed by Hojgaard et al. (2014) [30]. Briefly, 5 ul of DNA was assayed in a 15 ul reaction with 7.5 ul iQ Multiplex Powermix (BioRad), 1.5 ul 10X M2 primer mix (final concentration of forward and reverse primers = 300 nM, probes = 200 nM), and 1ul H_2_O. Negative and positive controls were used in all tests and performed in triplicate. For the latter, a plasmid containing PCR-amplified target genetic loci was generously provided by Andrias Hojgaard and Joseph Piesman. Pipetting to mix samples was performed in a PCR hood; positive control samples were set up in a separate lab to ensure no contamination of experimental sample DNA. PCR was carried out on a CFX 96 Touch Real-Time PCR Detection System (BioRad) using the following parameters: initial denaturation for 3 min at 95°C was followed by 40 cycles of 95°C for 10 sec and 60°C for 1 min.

To corroborate field identifications of *Peromyscus* spp., 63 DNA samples were selected for genetic analysis, including 12, 9, 24, and 18 samples corresponding to *Peromyscus* individuals captured at interior forest, natural edge, pasture edge, and residential edge habitat, respectively. The protocol for identification was adopted from Tessier et al. (2004) [31]. Two 10 ul PCR mixtures were prepared, one containing a forward primer specific to deer mice (*Peromyscus maniculatus*, F9197) and the other a forward primer specific to white-footed mice (*Peromyscus leucopus*, F9263). The reverse primer, H9375, was common among all PCR mixtures. Mixtures included 5 ul of 2X SSO Advanced SYBR Green Supermix, 3 ul H_2_O, 1ul template DNA, and 0.5 ul each of reverse and forward primers (final concentration of both primers was 500 nM). PCR was performed on a CFX 96 Touch Real-Time PCR Detection System (BioRad) using the following parameters: 98°C for 3 min, 30 cycles of 10 sec at 98°C, 30 seconds at 56°C and 20 sec at 72°C, and a final 2 min extension at 72°C. DNA from a known deer mouse museum specimen was used as the positive deer mouse control. Analyses confirmed that all *Peromyscus* captures were white-footed mice.

### Statistical analysis

To determine the effects of vegetation, edge type, and landscape characteristics on small mammal abundances, we used generalized linear models (GLM) with a Poisson error distribution. Dependent variables included total small-mammal abundance, white-footed mouse abundance, and northern short-tailed shrew abundance, with abundance being the total number of unique individuals captured at each site. Models were constructed *a priori* to test the hypotheses that white-footed mice would be more abundant in edge habitats with smaller patch sizes, while northern short-tailed shrews would respond positively to forest interiors and patch size. Abundances of masked shrew (*Sorex cinereus*), eastern chipmunk (*Tamius striatus*), and red squirrel (*Tamiasciurus hudsonicus*) were too low for analysis.

We modeled *B*. *burgdorferi*, *A*. *phagocytophilum*, and *B*. *microti* infection prevalence in all small mammals captured as a function of vegetation, edge type, and landscape characteristics using generalized linear models (GLM) with a binomial error distribution. We used a binary indicator of infection as a dependent variable. Models were developed *a priori* to test the hypotheses that infection would be greater in more fragmented areas with greater understory vegetation.

For all models, pairwise correlation coefficients were derived for independent variables to ensure that highly correlated variables (|r| > 0.7) were not included within the same model [32]. All independent variables were standardized to have similar scales and variances. Models were ranked using Akaike Information Criterion corrected for small sample size (AICc) [33]. We evaluated the strength of evidence for a model by comparing the AICc with an intercept-only null model, where all models with ΔAICc < 2.0 were considered as competitive models, and by determining if the 95% confidence intervals of the parameter estimates did not overlap with zero [33]. If the null model was the top model or had a ΔAICc < 2.0, none of the models were considered to be good predictors for the dependent variable of interest [33].

## Results

### Small-mammal communities

**Fig 2:**
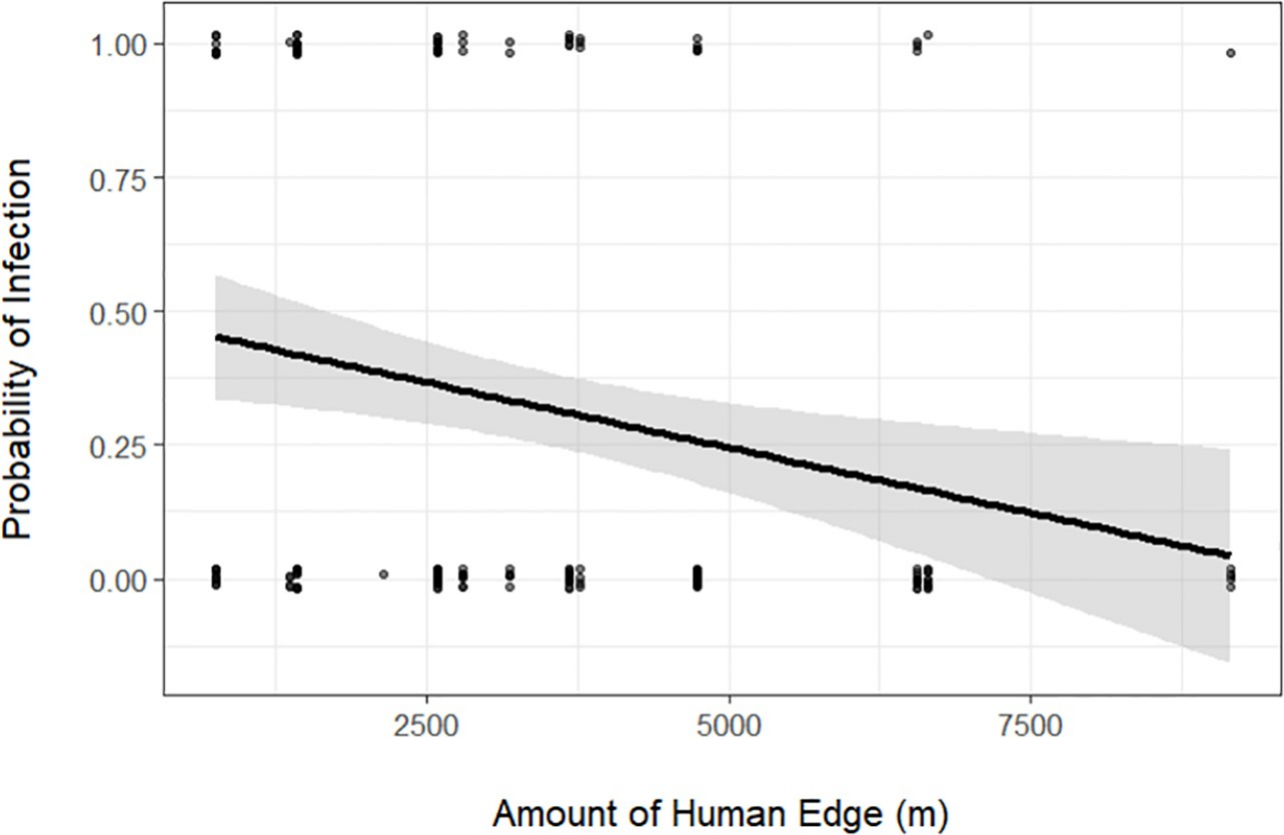
Individual abundance (mean ± SE) of white-footed mice (*Peromyscus leucopus*) and northern short-tailed shrews (*Blarina brevicauda*) in interior forest (INT), natural edge (NE), pasture edge (PE), and residential edge (RE) in northeastern Massachusetts during 2012 and 2013 (years combined).

The 2012 season yielded 103 unique individuals with a trapping success rate of 4.3%, whereas the 2013 season yielded 92 unique individuals with a trapping success rate of 3.8%. These 195 total unique captures represented 5 small-mammal species, including white-footed mouse (169 individuals), northern short-tailed shrew (18), eastern chipmunk (5), red squirrel (2) and masked shrew (1). No individual was recaptured at a different site. Only white-footed mice and northern short-tailed shrews were captured in large enough abundances for statistical analysis. White-footed mouse abundance was greatest at pasture edges (Fig 2); however, the competitive model including edge type was relatively low in weight. No models for northern short-tailed shrews included edge type.

Generalized linear models of small mammal abundance suggest species correlated to both vegetation and landscape characteristics. Abundance of white-footed mice was greater at study sites with less forested area in a 500-m radius (Table 1). Northern short-tailed shrew abundance was greater in fragmented landscapes at the 200-m radius, including positive association with fragmentation index, linear amount of human edge and wetland edge (Table 2). Shrew abundance also was positively related to abundance of shrubs and understory trees, although the relationship was relatively weak (weight = 0.02).

**Table 1:** Generalized linear models (GLM) with ΔAICc below the null model for vegetation and landscape measures for white-footed mouse abundance from 2012–2013 combined in northeastern Massachusetts.

White-footed Mouse Models	*K*	ΔAIC_c_	AIC_c_	ω_i_	LogLik
**Forest500**	**2**	**0**	**166.77**	**0.62**	**-81.18**
Patch Size+Forest500	3	2.06	168.84	0.22	-80.99
**Shrubs+Understory Trees**	**3**	**2.97**	**169.74**	**0.14**	**-81.44**
**Edge Type**	**4**	**8.69**	**175.46**	**0.01**	**-82.99**
Null Model	1	9.77	176.55	0.00	-83.53

Competitive models (ΔAICc ≥ 2.0 below the null model) are highlighted in bold. Numbers next to landscape metrics indicate the radius of measurement (m) for that variable.

**Table 2:** Generalized linear models (GLM) with ΔAICc below the null model for vegetation and landscape measures for northern short-tailed shrew abundance from 2012–2013 combined in northeastern Massachusetts.

Northern Short-tailed Shrew	*K*	ΔAIC_c_	AIC_c_	ω_i_	LogLik
**FragIndex200**	**2**	**0**	**38.83**	**0.45**	**-17.21**
FragIndex200+Patch_Size	3	0.57	39.40	0.34	-16.27
**Human200+Wetland200**	3	2.50	41.33	0.13	-17.24
**Shrubs+Understory Trees**	**3**	**5.24**	**44.08**	**0.03**	**-18.61**
Null Model	1	7.66	46.49	0.01	-22.18

Competitive models (ΔAICc ≥ 2.0 below the null model) are highlighted in bold, with their estimate and standard error. Numbers next to landscape metrics indicate the radius of measurement (m) for that variable.

### Infection prevalence

**Fig 3:**
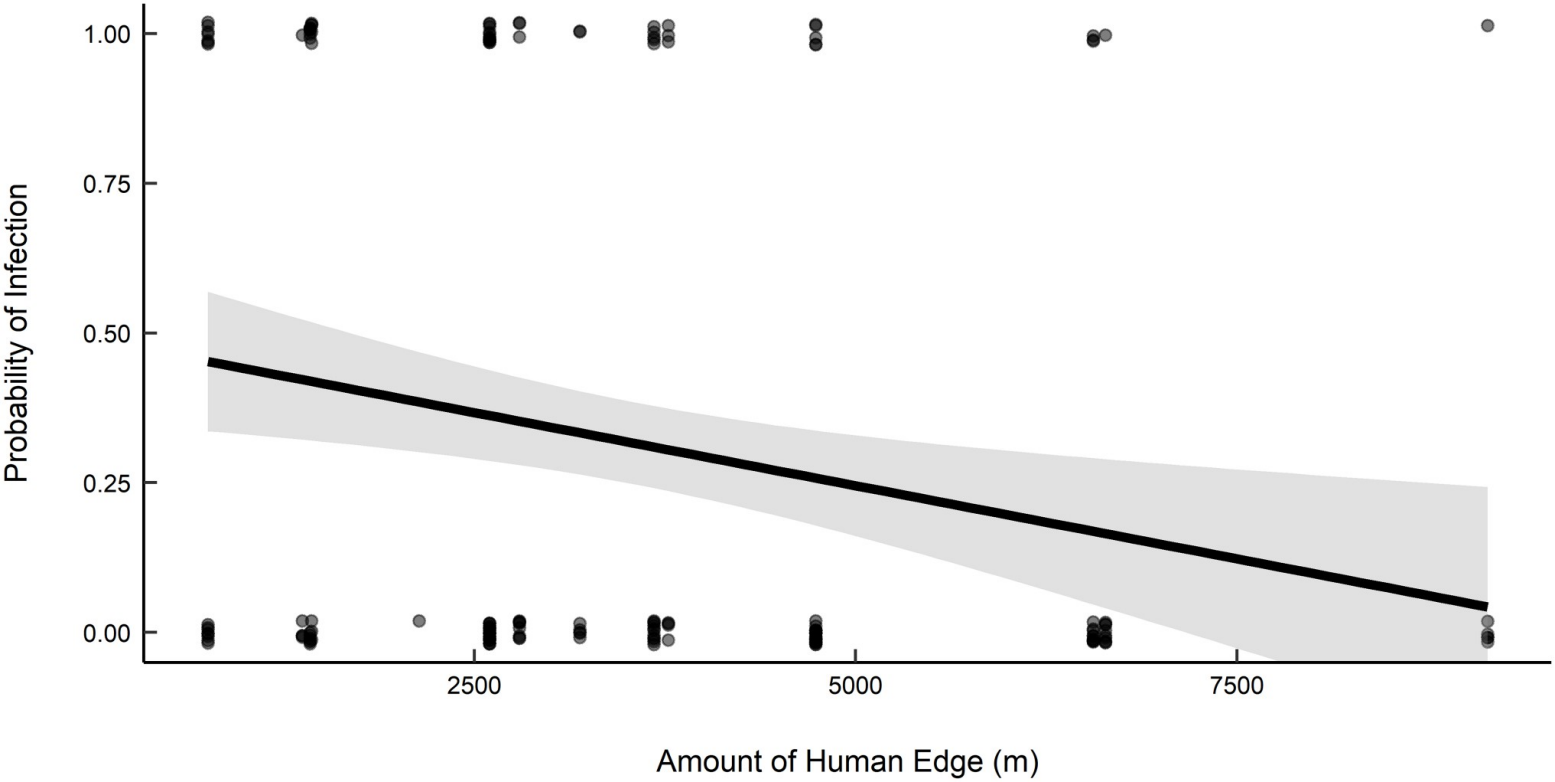
Probability of infection by *B*. *burgdorferi* in white-footed mice (*Peromyscus leucopus*) based on amount of human edge (m) within 200-m and 500-m combined radius of study sites in northeastern Massachusetts during 2012 and 2013 (years combined).

A total of 147 tissue samples from white-footed mice and 16 tissue samples from northern short-tailed shrews were analyzed for the presence of the *B*. *burgdorferi*, *A*. *phagocytophilum*, and *B*. *microti*. White-footed mouse infection prevalence for *B*. *burgdorferi* (total 32.7%) was greatest at interior-forest study sites compared to sites associated with a habitat edge (F = 3.48, df = 3,28, *P* = 0.03) at 16 study sites across two years (Table 3). Infection prevalence of *A*. *phagocytophilum* (mean infection = 5.4%) and *B*. *microti* (mean infection = 17.7%) did not differ significantly among edge types. Infection of northern short-tailed shrews with *B*. *burgdorferi* (mean infection = 6.3%), *A*. *phagocytophilum* (mean infection = 12.5%), and *B*. *microti* (mean infection = 25.0%) did not differ significantly among edge types. Most models for *B*. *burgdorferi* infection in white-footed mice included vegetation variables; we found a positive relationship between infection prevalence and presence of leaf litter and a negative relationship between infection prevalence and presence of vegetation ground cover (Table 4). At the landscape scale, we detected a combined relationship with edges for prevalence of *B*. *burgdorferi*, composed of a negative relationship with human edge (Fig 3) and positive relationship with wetland edge at the 500-m radius. We did not detect any competitive models for *A*. *phagocytophilum* or *B*. *microti*.

**Table 3:** *Borrelia burgdorferi*, *Anaplasma phagocytophilum*, and *Babesia microti* infection prevalence (#infected/#tested = %) in white-footed mice and northern short-tailed shrews for interior forest, natural edge, pasture edge, and residential edge in northeastern Massachusetts at 16 study sites during 2012 and 2013.

White-footed Mouse					
	Interior Forest	Natural Edge	Pasture Edge	Residential Edge	Total
Number tested	34	24	54	35	147
*B*. *burgdorferi*	15 (44.1%)^a^	9 (37.5%)^b^	16 (29.6%)^b^	8 (22.9%)^b^	48 (32.7%)
*A*. *phagocytophilum*	2 (5.9%)	1 (4.2%)	4 (7.4%)	1 (2.9%)	8 (5.4%)
*B*. *microti*	8 (23.5%)	2 (8.3%)	11 (20.4%)	5 (14.3%)	26 (17.7%)
Northern Short-tailed Shrew					
Number tested	0	3	4	9	16
*B*. *burgdorferi*	0	0 (0.0%)	0 (0.0%)	1 (11.1%)	1 (6.3%)
*A*. *phagocytophilum*	0	0 (0.0%)	0 (0.0%)	2 (22.2%)	2 (12.5%)
*B*. *microti*	0	1 (33.3%)	2 (50.0%)	1 (11.1%)	4 (25.0%)

For significant differences, habitats with percentages with the same superscript for a given infection are not significantly different from each other.

**Table 4:** Generalized linear mixed-effect models for vegetation and landscape metrics for *Borrelia burgdorferi* s.s. prevalence for white-footed mice (*Peromyscus leucopus*) at all sites combined in northeastern Massachusetts during 2012 and 2013.

*Borrelia burgdorferi s*.*s*.	*K*	ΔAIC_c_	AIC_c_	ω_i_	LogLik
**Leaf Litter**	**2**	**0**	**209.74**	**0.34**	**-102.83**
**Veg Cover**	**2**	**0.82**	**210.55**	**0.22**	**-103.24**
**Wetland500+Human500**	**3**	**1.82**	**211.56**	**0.14**	**-102.71**
TBA Conifer	2	3.73	213.46	0.05	-104.70
TBA Decid+Leaf litter+Canopy	4	4.12	213.86	0.04	-102.81
Wetland200+Human200	3	4.37	214.11	0.04	-103.98
Shrubs+Understory	3	4.38	214.11	0.04	-103.98
TBA Conifer+Conifer Density	3	5.34	215.08	0.02	-104.47
Null Model	1	5.58	215.31	0.02	-106.65

Competitive models (ΔAICc ≥ 2.0 below the null model) are highlighted in bold. Numbers next to landscape metrics indicate the radius of measurement (m) for that variable. Prevalence of *Anaplasma phagocytophilum* and *Babesia microti* did not have competitive models and are not shown.

## Discussion

We found that white-footed mice and northern short-tailed shrews in northeastern Massachusetts were more abundant in fragmented landscapes at the 500-m and 200-m radii scales, respectively. We also documented a significantly greater prevalence of *B*. *burgdorferi* in white-footed mice in interior forest, indicating differential degrees of exposure in different edge types. Furthermore, we found greater infection prevalence associated with more leaf litter and vegetation cover at fine scales, and more wetland edge at the 500-m radius scale.

### Habitat associations

We found that abundances of white-footed mice and northern short-tailed shrews were dependent on factors from multiple scales, including fine-scale vegetation characteristics, edge type, and broader-scale landscape characteristics. Shrew populations, in particular, were influenced across the full continuum of scales considered, from local shrub and understory tree density to the human-induced and natural wetland edges at a 200-m radius. We found that white-footed mouse abundance was positively correlated with shrub cover at fine scales and negatively associated with human-induced edge within a 500-m radius. Previous studies on white-footed mice habitat associations in Massachusetts have produced conflicting results, where abundances have been shown to increase across woody gradients [34] but densities decrease with herbaceous stem density [35].

Induced edges generate greater habitat heterogeneity at the interface due to natural succession and the dynamics of novel edge effects [36]. Increased vegetation diversity at the induced edge may accommodate a greater number of habitat requirements for different species. In our study, we did not find an induced-edge relationship in abundance of northern short-tailed shrews, similar to Sekgororoane and Dilworth (1995) [37], who also detected no affinity for induced edges. Given the preference by the northern short-tailed shrew for moist environments [38, 39], we expected edge avoidance and greater abundance in interior forest with a more humid microclimate or greater abundance at natural edges associated with wetlands. Many residential-edge sites were adjacent to lawns or garden beds and may have been influenced by moisture from nearby irrigation and watering, resulting in an increased abundance at these sites.

In contrast, although a well-documented generalist, the white-footed mouse exhibited greater abundance at pasture edges, although the weight of this model was low. Anderson et al. (2003) [40] documented greater densities in small compared to large patches, likely a result of higher captures at edge interfaces due to greater structural complexity. However, other researchers have reported no difference in densities [41] or lower densities at forest edges [42, 43]. These patterns may indicate true differences in habitat quality [7, 36], but also discrepancies in arbitrary edge definition between studies [44]. However, one limitation of our study is that without long-term demographic data, our observations represent a snapshot of small-mammal abundances.

Infection prevalence of *B*. *burgdorferi* in white-footed mice was greater at forest-interior sites than at any edge-habitat site, while *A*. *phagocytophilum* and *B*. *microti* were not associated with any vegetation or edge characteristics. Greater *B*. *burgdorferi* infection prevalence in forest interiors may be a function of increased reservoir abundance [16] although small-mammal abundance was intermediate at forest interiors compared to edges in our study. Other possible reservoirs that may account for this effect include ground-dwelling birds, such as American robins (*Turdus migratorius*), or small mammals that are difficult to capture in Sherman traps, such as eastern chipmunks [13]. Additionally, the similarities of interior-forest habitat to the other edge types in terms of vegetation characteristics make it difficult to attribute differences to vegetation structure and complexity, such as the relationship we found with leaf litter and vegetative cover relative to *Borrelia* prevalence. Further analysis and sampling is required to establish the exact mechanism of this effect.

### Fragmentation effects

The presence of induced edge and reduced forested habitat are closely associated with fragmentation processes, indicating that our results support an implicit positive relationship between small-mammal abundance and habitat fragmentation [45]. Although we found only a limited difference in white-footed mouse and northern short-tailed shrew abundance based on edge type, we did find that both mice and shrews were positively affected by fragmentation measures at the landscape scale. Specifically, mice were more abundant at sites with decreasing forested area at the 500-m radius, and shrews were more abundant at sites with a higher fragmentation index (greater induced edge to forest ratio) and with greater human and wetland edge at the 200-m radius. These results counter some of the directional trends found in the literature, which indicate that small-mammal abundance is negatively related to fragmentation indices, such as smaller remnant patches and increased forest management activity [6, 46]. Differences in ecosystem context [46] or community composition [6] may account for the varied impacts of fragmentation on our small-mammal communities. Debinski and Holt (2000) [4] and Bowers and Matter (1997) [47] argue that variations in abundance in fragmented landscapes among studies can also be explained by temporal (age of remnant patch) and spatial (isolation of patches) variables, neither of which were quantified in this research. Both white-footed mice and northern short-tailed shrews may benefit from the land-use heterogeneity created by fragmentation [34]. This pattern links well with the findings of Nupp and Swihart (1996) [7], who found that small forest patches in Indiana had higher densities of white-footed mice compared to larger patches and contiguous forest.

We found a negative relationship between *B*. *burgdorferi* prevalence and habitat fragmentation indices. General trends in the literature suggest that *B*. *burgdorferi* infection probabilities are correlated with white-footed mouse habitat preferences, where infection prevalence increases in fragmented landscapes. This pattern has been shown in black-legged ticks, where tick density and *B*. *burgdorferi* infection prevalence tend to increase with decreasing patch size and increasing patch isolation [18, 19]. Our results, based on prevalence in white-footed mice instead of black-legged ticks, contradict this pattern. Additionally, infection prevalence was greatest in interior forest and decreased as linear amount of human edge increased, while increasing with natural heterogeneity of wetland edge at the 500-m radius. Linske et al. (2018) [48] found that *B*. *burgdorferi* infection in white-footed mouse was significantly higher in large forest patches compared to forest edges. They attributed this finding to the dilution effect, where edge habitats had greater encounter probabilities with alternative hosts. We did not directly test for the dilution effect as we did not estimate the abundances of alternative reservoirs to small mammals and we did not measure tick infestations on small mammals. However, the dilution effect is one possible explanation for our observations.

In contrast to findings for *B*. *burgdorferi*, significant models were not identified for infection prevalence for *A*. *phagocytophilum* and *B*. *microti* based on edge type or landscape characteristics. Other researchers have shown that *B*. *microti* prevalence in humans responds to amount of landscape-level edge habitat [49], but that *A*. *phagocytophilum* prevalence, based on black-legged tick infection, is not related to landscape-level composition [20]. However, limited research has been conducted on these pathogens. Additional studies are required to understand patterns of infection and co-infection [50] of these pathogens related to particular vegetation and landscape features. In addition, although infection by *B*. *microti* is typically detected through blood collection rather than tissue collection [51, 52], relatively high rates of infection (18.4% for *P*. *leucopus* and *B*. *brevicauda* combined) in our study suggest that collection of an ear or tail sample is a viable alternative to detect this pathogen, given the vascularization of this tissue. However, this comparison requires more direct analysis; lack of significant differences based on vegetation, edge type, or landscape characteristics may indicate an underestimate of this pathogen in our study.

## Conclusions

Overall, we found multiscale correlations of vegetation, edge type, and landscape variables on small-mammal abundance and *B*. *burgdorferi* prevalence, emphasizing the importance of scale considerations in the ecology of small-mammal communities and disease transmission. In addition, this relationship between small-mammal infection prevalence and patch interiors is rare in the body of relevant literature and offers novel insight into the ecology of Lyme disease in the northeastern U.S. However, given small sample sizes in this study, additional research is required to corroborate these results and to help identify patterns for other disease pathogens for which small mammals are the common reservoir, including *A*. *phagocytophilum* and *B*. *microti* [53].

## Supporting information

S1 File Final mammal infectivity data with GIS and vegetation. (XLSX)
